# Effect of Electroacupuncture at GV20 on Sleep Deprivation-Induced Depression-Like Behavior in Mice

**DOI:** 10.1155/2020/7481813

**Published:** 2020-08-21

**Authors:** Xiaohong Xu, Peng Zheng, Hongyan Zhao, Bailin Song, Fuchun Wang

**Affiliations:** ^1^Changchun University of Chinese Medicine, Changchun 130117, China; ^2^Jilin Provincial Hospital of Traditional Chinese Medicine, Changchun University of Chinese Medicine, Changchun 130021, China; ^3^Institute of Basic Theory of Chinese Medicine, China Academy of Chinese Medical Science, Beijing 100700, China

## Abstract

Accumulating evidence suggests that sleep deprivation (S-Dep) is a critical risk factor for depression. Electroacupuncture (EA) treatment has been reported to ameliorate posttraumatic stress disorder- (PSTD-) like behavior and enhance hippocampal neurogenesis. However, whether EA treatment has any beneficial effect on S-Dep-induced depression-like behavior is still unknown. In the present study, we focused on whether EA at Baihui (GV20) can ameliorate the deterioration effect of S-Dep in mice. Mice were randomly divided into normal, S-Dep, S-Dep + EA, and S-Dep + sham EA groups. Cognitive behavior test and in vitro assay were performed separately to avoid the influence of behavior test on synaptic transmission and protein expression. Depression-like behaviors were determined by forced swimming test (FST), tail suspension test (TST), and Morris water maze (MWM). Neurogenesis was identified by BrdU, DCX, and NeuN immunofluorescence staining. In vitro long-term potentiation was detected by high frequency stimulation (HFS) at Schaffer collateral-CA1 synapses in hippocampal slices. Brain-derived neurotropic factor (BDNF) and tropomyosin receptor kinase B (TrkB) protein expression level were assayed by western blot. Our results indicated that D-Sep mice demonstrated depression-like behaviors determined by prolonged immobility duration in FST and TST; D-Sep mice also manifested spatial memory retention deficit in MWM. Furthermore, EA treatment ameliorated D-Sep-induced depression-like behaviors and spatial memory retention deficit. Mechanically, EA treatment alleviated neuron progenitor cell proliferation and differentiation, ameliorated the field excitatory postsynaptic potentials (fEPSPs) slope impaired by S-Dep, and elevated BDNF/TrkB protein expression. Taken together, our data suggested that EA treatment has a protective effect on S-Dep-induced depression-like behavior and cognitive impairment, which may be through regulating BDNF/TrkB protein expression.

## 1. Introduction

Sleep, which has been discovered as a state of consolidation of newly formulated memory, is crucial for learning new information and mental performance [1]. Sleep loss or insomnia has been proven to contribute to hippocampal-dependent cognition deficits and depression-like behavior; the underlying mechanisms include disruption of synaptic plasticity at electrophysiology and molecular levels as well as at a structural level [2]. Long time potentiation (LTP) is a form of synaptic plasticity adopted as an in vitro biological model of learning and memory. Ample evidence has demonstrated that sleep deprivation can have detrimental effects on memory formation and LTP induction [3–5]. BDNF, a member of the neurotrophin family, is a significant modulator in synaptic plasticity and memory formation. BDNF needs to bind to its high-affinity protein kinase receptor, TrkB, to exhibit its biological effect [6].

Acupuncture, a traditional therapy originating from ancient China, is an alternative and complementary medicine that has long been known to have therapeutic effect on various neurodegenerative diseases including depression [7]. Abundance studies indicated that acupuncture or electroacupuncture treatment ameliorates cognitive impairment and improves hippocampal-dependent synaptic plasticity. GV20 is located above the apex auriculate, on the midline of the head. Clinically, acupuncture at GV20 has been widely used in neuropsychological diseases including depression, anxiety, Parkinson's disease, and neurasthenia, although the underlying mechanism is still not fully illustrated. Acupuncture stimulation at GV20 has been proven to improve scopolamine-induced cognitive impairment and alleviate BDNF expressions [8]. A former study demonstrated that acupuncture at GV20 produced instant sedative effect and altered electroencephalogram *α* and *β* waves frequencies in a model of sleep deprivation [9]. EA at GV20 and Shenting (GV24) attenuated the expression of monoamine neurotransmitters and IL-1 beta in the hypothalamus after sleep deprivation in rats with focal cerebral ischemia/reperfusion injury [10].

EA treatment or pretreatment has been shown to modulate ESPS-induced anxiety-like behaviors and prevent hippocampal neurogenesis disruption in a rat model of PTSD [11, 12]. However, the effects of EA at GV20 on S-Dep-induced hippocampal synaptic plasticity impairment and depression-like behavior have remained elusive. The present study investigated the effects of EA at the GV20 acupoints on S-Dep mice by depression-related behavior test, neurogenesis detection, LTP induction, and BDNF/TrkB signaling protein assay.

## 2. Materials and Methods

### 2.1. Animals

**Figure 1:**
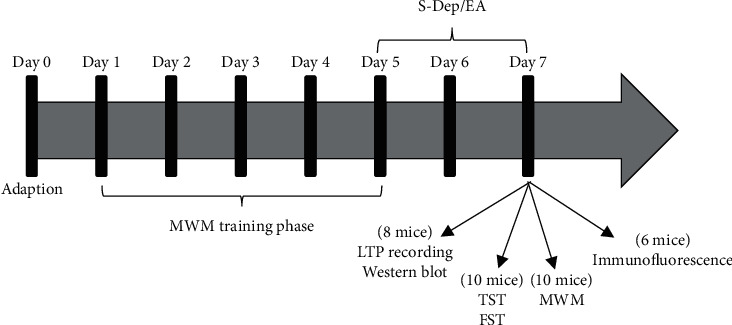
Schematic illustration of the experimental procedure. All mice were assigned into four groups: normal, S-Dep, S-Dep + EA, and S-Dep + sham EA group. A set of mice containing 10 mice were exposed to MWM training phase test during 5 consecutive days, followed by 48 h of S-Dep and EA or sham EA treatment once daily; on the 7th day after the last EA treatment, mice were used for exploring phase test. Another set of mice containing 10 mice underwent TST and FST; 3 h recovery time was given after TST. Two separated sets of mice from each group were sacrificed after S-Dep and EA/sham EA treatment for LTP/western blot or immunofluorescence.

Male BALB/c mice aged 2–3 month were obtained from the Experimental Animal Research Center of Hubei (Wuhan, China). The animals were housed in a 12 h light-dark cycle (07 : 00–19 : 00) and had free access to food and water. The room temperature was maintained at 23 ± 1°C. All mice were adapted to the environment for 1 week before the experiment. The mice were randomly divided into 4 groups: normal group, S-Dep group, S-Dep + EA group, and S-Dep + sham EA group, with each group containing 34 mice (Figure 1). The normal group did not receive any treatment but were immobilized by hand with gentle plastic restraints just as the treatment groups. After S-Dep and EA treatment, a subset of animals were used for behavior test (*n* = 10/group), LTP induction (*n* = 8/group), and biochemical analysis (*n* = 6/group). All experimental procedures complied with the guidelines of the Principles of Laboratory Animal Care and the legislation of the People's Republic of China for the Use and Care of Laboratory Animals.

### 2.2. Sleep Deprivation Model

The multiple-platform apparatus was adopted to establish sleep deprived mice as previously described [13]. In short, animals were placed in a chamber with multiple small platforms (diameter: 3.5 cm) which was 1 cm above water. When animals entered rapid eye movement (REM) sleep, their muscle tone diminished and the animals fell into the water, waking them up and preventing them from going to sleep. For normal mice, the small platforms were replaced with larger ones (diameter: 13 cm), allowing them to enter REM sleep without falling into water. S-Dep was induced for 48 h in this experiment.

### 2.3. Electroacupuncture Stimulation

EA treatment was taken once daily during the 48 h of S-DEP. The EA stimulus was continued for 30 min with insertion to a depth of 5 mm at Baihui (GV 20). A previous study had found that EA at the intensity of 2 mA and 100 Hz (pulse width: 0.2 ms, duration: 10–30 min) has significant analgesic effect in mice [14]; in light of this study, biphasic square pulse (2 mA, 0.2 ms) with high frequency, 100 Hz, was administered through a medical EA apparatus (Qingdao Xinsheng instrument, Qingdao, China). The needles (0.25 mm × 25 mm) were purchased from Suzhou Hualun Medical Appliance Co., Ltd. (Suzhou, China). In the Sham EA treatment group, acupuncture needles were inserted superficially into the acupoints without electrical stimulation. At the end of the last EA treatment, mice were subjected to behavioral, electrophysiological, and immunochemical assays separately.

### 2.4. Tail Suspension Test

TST was used to analyze depressive-like behavior as previously described [16]. A short piece of paper adhesive tape (about 6 cm) was attached along half the length of the tail (about 3 cm). The mouse's head was about 20 cm above the floor. The test was carried out for 6 min, during which mouse immobility time was recorded, with the absence of initiated movements defined as immobility. The first 2 min activity was considered pretreatment period, and the duration of immobility was video-recorded during the final 4 min.

### 2.5. Forced Swimming Test

Three hours after the TST, mice were placed in a cylinder container (diameter, 25 cm; height, 50 cm) containing fresh water (temperature 24 ± 1°C) for 6 min, and behavior activity was video-recorded. The first 2 min was considered to be pretest swim and was excluded from the analysis. Immobility duration of the last 4 min was counted.

### 2.6. Morris Water Maze

MWM was adopted to evaluate spatial-related working memory as previously described [15]. The experiments were conducted in a tank (124 cm in diameter) filled with water (32 cm in depth). The water was made opaque by white nontoxic paint, and temperature was kept at 22 ± 1°C during the experiment. Mice were trained to find a cylindrical platform (10 cm in diameter). The MWM test was divided into two phases, the training phase and the exploring phase. Each mouse performed four trials daily for 5 days during the training phase; the mice were allowed to swim for 60 s to find the hidden platform. If mice successfully reached the platform within 60 s, they were allowed to stay there for 15 s, if they failed, they were manually put on the platform. The exploring test was conducted on the 7th day after the last EA treatment while the platform was removed. The traces of exploring, time spent in the target quadrant, and number of times of crossing platform in the exploring session were analyzed.

### 2.7. LTP Recording

Eight mice from each group were sacrificed after final EA treatment. Brains were immediately removed and placed in ice-cold oxygenated artificial cerebrospinal fluid (ACSF) containing (in mM) 100 sucrose, 60 NaCl, 4 KCl, 8 MgCl_2_, 1.3 NaH_2_PO_4_, 30 NaHCO_3_, 5 D-glucose, and 0.5 CaCl_2_ (pH 7.2–7.4) saturated with carbogen (95% O_2_−5% CO_2_). The left side of brains was placed in liquid nitrogen for the western blot, and the right side was used for LTP recording. Coronal hippocampal slices (300 *μ*m) were cut using a vibratome (VT1000S, Leica, Germany) in ice-cold oxygenated cutting solution. Recording solution contained (in mM) 125 NaCl, 2 CaCl_2_, 2.5 KCl, 1 MgCl_2_, 25 NaHCO_3_, 1.25 NaH_2_PO_4_, and 25 D-glucose (pH 7.2–7.4). LTP experiment was conducted using a bipolar stimulating electrode, stimulus electrode was located at Schaffer collateral, and recording electrode was positioned at radium stratum in CA1. The stimulus intensity was set to evoke 40–50% of the maximal amplitude of fEPSPs. fEPSPs were recorded every 30 s, and LTP was induced by a tetanic stimulus at 100 Hz for 2 s. Baseline responses were recorded for 20 min, followed by the continued recording of the fEPSPs for 60 min.

### 2.8. Immunofluorescence

After the finial EA treatment, 6 mice from each group were sacrificed and perfused with PBS followed by 10% formalin. Then the brain samples were cut into 20 *μ*m slices coronally. From each brain, three slices were selected including the DG of hippocampus for staining. The slices were blocked by 0.3% BSA and 0.3% Triton X-100 to block nonspecific binding, then incubated with primary antibody of BrdU, NeuN, and doublecortin (DCX) for 24 h at 4°C, followed by incubating with secondary antibody of Alexa Fluor 488 donkey anti-rabbit IgG (1 : 1000, Invitrogen, Thermo Fisher Scientific) and Alexa Fluor 594 goat anti-mouse IgG (1 : 1000, Invitrogen, Thermo Fisher Scientific) for 2 h at room temperature. Images were captured under a fluorescence microscope (ECLIPSE 90i, Nikon, Japan).

### 2.9. Western Blot Analysis

The hippocampus tissues were isolated and lysed in 100 *μ*l Radioimmunoprecipitation Assay (RIPA) buffer (Thermo Fisher Scientific, Waltham, MA) plus protease inhibitors. The protein concentration was quantified by the Bicinchoninic Acid Protein Assay Kit (Beyotime Biotechnology, Shanghai, China). Total protein (50 *μ*g) was processed for 12% SDS-PAGE and transferred onto PVDF membranes (Millipore, Billerica, MA). Blots were blocked by 5% nonfat milk and immunoblotted with anti-BDNF (1 : 2000, Abcam), anti-TrkB (1 : 300, Cell Signal), and anti-GAPDH (1 : 1000, Abcam). The membranes were incubated with the horseradish peroxidase-conjugated secondary antibody for 1 h at room temperature. The protein bands were detected using ECL and analyzed by Image-Pro Plus 6.0.

### 2.10. Statistical Analysis

GraphPad Prism 7.0 software was used to perform all analyses. Data are presented as mean ± SD. Data were analyzed using two-sample Student's *t*-test for two group comparisons, and two-way analysis of variance (ANOVA) was conducted for comparison between multiple groups. The significance threshold was set to *p* values < 0.05.

## 3. Results

### 3.1. EA Reversed Depression-Like Behavior Induced by S-Dep

**Figure 2:**
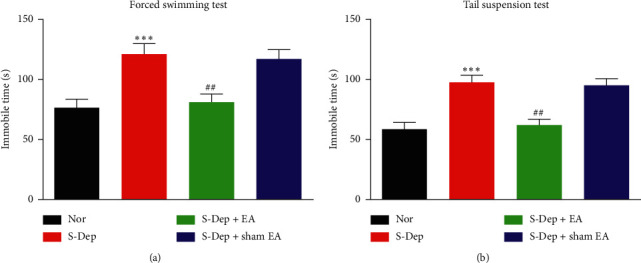
EA reversed depression-like behavior induced by S-Dep. The immobility time on FST (a) and TST (b). Data were presented as the mean ± SD, *n* = 10; ^*∗∗∗*^*p* < 0.001 vs. normal group; ^##^*p* < 0.01 vs. sleep deprivation group.

FST and TST were both classic behavior test models for depression-like symptoms. The immobility time in FST was markedly elevated in S-Dep group (121.50 ± 8. 94 s) compared with normal group (76.56 ± 7. 12 s), which was reversed by EA treatment (81.35 ± 6. 95 s). However, sham EA treatment (117.36 ± 7. 91 s) has no significant influence on the immobility time compared with the S-Dep group (Figure 2(a), all *p* < 0.01). In accordance with the FST results, the immobility time in TST was significantly increased in the S-Dep group (97.58 ± 6. 15 s) and sham EA group (95.20 ± 5. 74 s) compared with the normal group (58.64 ± 5.71 s); EA treatment (62.12 ± 4. 93 s) counteracted S-Dep induced rise of immobility time (Figure 2(b), all *p* < 0.01). These data suggest that EA treatment ameliorates S-Dep-induced depression-like behavior.

### 3.2. EA Mitigated Spatial Memory Retention Deficits Induced by S-Dep

**Figure 3:**
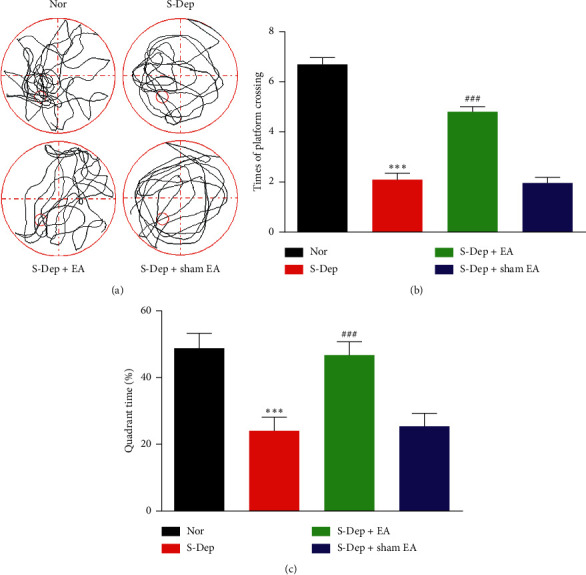
EA mitigated spatial memory retention deficits induced by S-Dep. The spatial memory retention of mice was determined by MWM. (a) Representative traces of activity of mice in the MWM. Number of times of platform crossing (b) and percentage of time spent in target quadrant (c) during the exploring phase. Data were presented as the mean ± SD, *n* = 10; ^*∗∗*^*p* < 0.001, ^*∗∗∗*^*p* < 0.001 vs. normal group; ^##^*p* < 0.01, ^###^*p* < 0.001 vs. sleep deprivation group.

We further investigated the effect of EA treatment on spatial memory retention by MWT. The traces of activity by mice were shown in Figure 3(a). Spatial memory retention ability was significantly impaired by S-Dep, as indicated by increased times of crossing the platform (Figure 3(b), *p* < 0.001) and less time spent in the target quadrant (Figure 3(c), *p* < 0.001) in the exploring phase. However, EA treatment dramatically ameliorated the memory retention ability of S-Dep-induced mice. Compared with S-Dep group, times of crossing the platform were attenuated, and time spent in the target quadrant was mitigated. Taken together, EA treatment protect against spatial memory retention deficit caused by S-Dep in mice.

### 3.3. EA Alleviated Neuron Progenitor Cell Differentiation in the DG of Hippocampus

**Figure 4:**
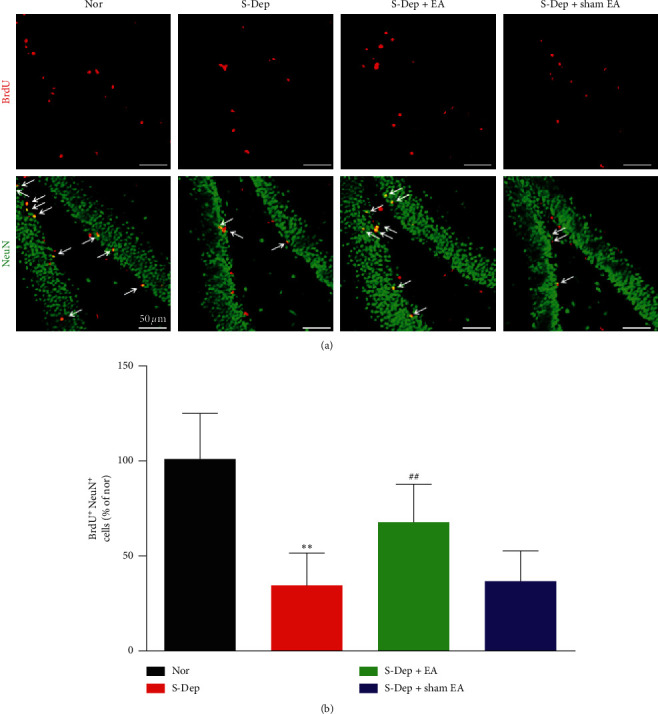
EA alleviated neuron progenitor cell differentiation in the DG of hippocampus. (a) Representative microphotograph of BrdU (red) and NeuN (green) double immunostaining showing matured newborn neurons in the DG. (b) Quantification of BrdU and NeuN double positive cells (yellow). Data were presented as the mean ± SD, *n* = 6; ^*∗∗∗*^*p* < 0.01 vs. normal group; ^##^*p* < 0.01 vs. sleep deprivation group.

To determine the effect of EA treatment on neurogenesis in S-Dep induced mice, we carried out immunofluorescence staining experiment. BrdU is a marker of proliferation cells, and NeuN was used to label the mature neurons. As is shown in Figure 4(a), BrdU and NeuN double positive cells were dramatically attenuated in the D-Sep group but were elevated in the EA treatment group, while sham EA treatment had no influence on BrdU and NeuN double positive cell number (Figure 4(b), all *p* < 0.01). The above results indicated that EA treatment increased the neuron progenitor cells differentiated into neurons.

### 3.4. EA Alleviated Neuron Progenitor Cells Proliferation in the DG of Hippocampus

**Figure 5:**
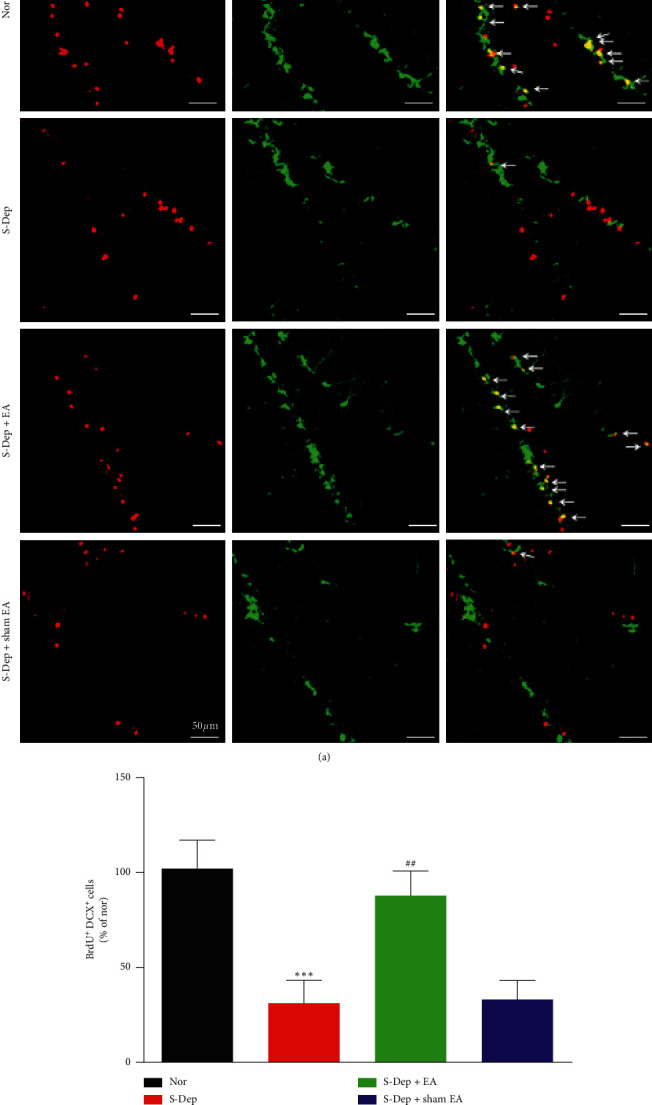
EA alleviated neuron progenitor cell proliferation in the DG of hippocampus. (a) Representative microphotograph of BrdU (red) and DCX (green) double immunostaining showing immature neurons in the DG. (b) Quantification of BrdU and DCX double positive cells (yellow). Data were presented as the mean ± SD, *n* = 6; ^*∗∗*^*p* < 0.01 vs. normal group; ^##^*p* < 0.01 vs. sleep deprivation group.

To further determine the effect of EA treatment on neurogenesis, BrdU and DCX double immunofluorescence staining were conducted in DG of hippocampus. DCX was widely adopted to label immature cells. As is shown in Figure 5(a), S-Dep attenuated BrdU and DCX double positive cell expression, which was reversed by EA treatment, but not altered by sham EA treatment (Figure 5(a), all *p* < 0.01). This study demonstrated that EA treatment facilitated neuron progenitor cell self-renewal and proliferation.

### 3.5. EA Abolished LTP Impairment Induced by S-Dep

**Figure 6:**
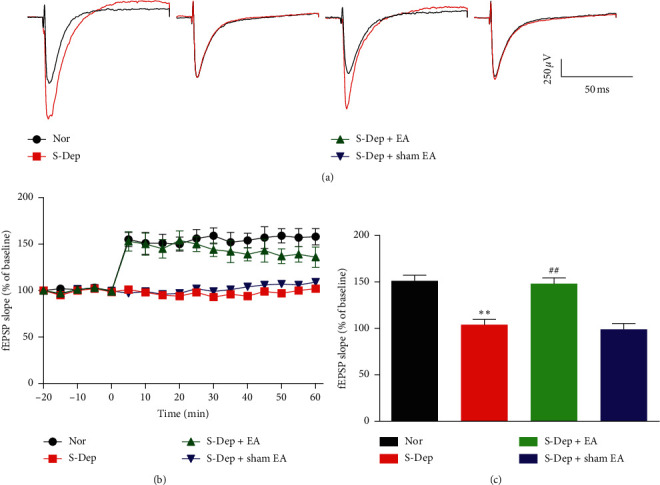
EA abolished LTP impairment induced by S-Dep. (a) Representative traces of the fEPSPs in the CA1 region of hippocampus before and after HFS in the Schaffer collaterals. (b) Plot of fEPSPs slope values as a percentage of baseline. (c) fEPSPs slope values were averaged from the duration 50–60 min after HFS. Data were presented as the mean ± SD, *n* = 8; ^*∗∗*^*p* < 0.01 vs. normal group; ^##^*p* < 0.01 vs. sleep deprivation group.

To determine the effect of EA on learning and memory in synaptic transmission level, hippocampal Schaffer collateral-CA1 LTP was recorded. The fEPSPs slope was significantly impaired in the S-Dep group compared with the normal group during the period of 10–60 min after HFS, which was abolished by EA treatment, but was not changed by sham EA treatment (Figures 6(a) and 6(b), all *p* < 0.05). The mean fEPSPs slope value at the period of 50–60 min in the S-Dep group (105.68 ± 8. 95) was significantly lower than normal group (152.50 ± 9. 34) and the EA treatment group (148.72 ± 8. 83) (Figure 6(c), all *p* < 0.01). The above data suggest that EA treatment ameliorated LTP deterioration by D-Sep.

### 3.6. EA Reversed BDNF/TrkB Expression in S-Dep Mice

**Figure 7:**
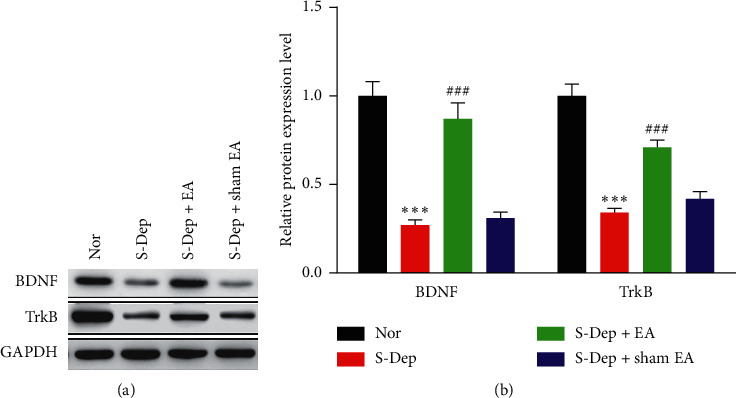
EA reversed BDNF/TrkB expression in S-Dep mice. Expression of BDNF and TrkB in hippocampal CA1 region was detected by western blot. (a) Western blot bands of BDNF and TrkB; GAPDH was used as an internal control. (b) Quantified protein expression level of BDNF and TrkB. Data were presented as the mean ± SD, *n* = 8; ^*∗∗∗*^*p* < 0.001 vs. normal group; ^###^*p* < 0.001 vs. sleep deprivation.

To investigate the molecular mechanism underlying protective effect of EA on S-Dep-induced cognitive impairment, western blot was conducted to detect protein expression in the hippocampus. The protein expression level of BDNF and TrkB was dramatically mitigated by S-Dep, while being obviously boosted by EA treatment. There were no significant differences in protein expression level of BDNF and TrkB in sham EA treatment group (Figures 7(a) and 7(b), all *p* < 0.001). Collectively, EA treatment abolished S-Dep-induced reduction of BDNF/TrkB protein level.

## 4. Discussion

Acupuncture treatment has been proven to exert antidepression effects on insomnia patients and can be safely applied in insomnia treatment [17]. Stimulation at GV20 located at the top of the head is widely accepted for the cure of neuropsychiatric disorders in the clinical practice, however, the underlying molecular mechanism is controversial. The current study uses a mice model of sleep deprivation to investigate the potential mechanism of EA at GV20 acupoints on cognitive impairment. Consistent with previous studies [18], our research suggested that S-Dep for 48 h induced depression-like behavior as shown by decreased immobility time in FST and TST. After EA treatment for 2 consecutive days, the immobility durations during the FST and TST in the S-Dep + EA group were significantly lower than those of the S-Dep group, indicating an antidepression effect of EA. Further, the spatial memory retention ability was estimated by MWM. The fewer times of crossing the platform and less time spent in the target quadrant suggest a significant amelioration of spatial memory retention impairment in the S-Dep + EA group compared with the S-Dep group. In contrast, the S-Dep + sham EA group produced no significant protective effects compared with the S-Dep group. All these data suggest that EA at GV20 has a protective effect on S-Dep-induced depression. A study using a p-chlorophenylalanine-induced insomnia rat model has indicated that EA treatment facilitates melatonin secretion [19], which exerts a significant antidepression effect. Another study reported that acupuncture treatment at GV20 has a sedative effect, increases *α* wave frequency, and decreases *β* wave frequency in rats after 72 h of sleep deprivation [20]. Interestingly, a previous study using 72 h sleep deprivation rat model indicated that EA at GV20 and ST36 simultaneously improved memory deficit [21], which is in accordance with our results using 48 h sleep deprivation mice model while using EA at GV20 alone. In comparison, our data extended the beneficial effect of EA for S-Dep from rat to mice and proved that EA at only acupoint GV20 could achieve this effect. Collectively, we concluded that EA at GV20 has a protective effect on S-Dep-induced depression. It is worth noting that the stimulus intensity we used in the current study resulted in a manifestation of gentle head nodding, indicating that the stimulation is well-tolerated by mice. More importantly, there is no significant behavior alteration in the first hour after EA. The amelioration of behavior test by EA appeared one hour after stimulation; this delay in EA effect may suggest that it takes time for EA treatment to regulate neural function and network activity. Thus, we speculated that EA amelioration of sleep deprivation-induced depression-like behavior is dependent on the GV20 acupoints, rather than direct electroconvulsive shock.

Hippocampus neurogenesis has been well-documented to play a pivotal role in spatial-related memory formation and depression-like behavior; thus, we performed immunofluorescence staining to evaluate the BrdU-labeled proliferating cell and DCX-labeled immature cell expression. BrdU-positive neurons and DCX-positive cells were markedly mitigated in the S-Dep group compared with the normal group but were significantly elevated in the S-Dep + EA group. The above results demonstrated that EA treatment facilitates neuron progenitor cell proliferation and differentiation in S-Dep mice. EA at GV20 and GV14 has been reported to improve neurogenesis after stroke [22]. Taken together, we demonstrated that neurogenesis evolved in the beneficial effect of EA treatment in cognitive impairment.

LTP, a long-lasting enhancement of synaptic transmission efficacy, is widely adopted as an electrorheological mechanism for storage of learning and memory [23, 24]. Our results showed that HFS-induced potentiation of the fEPSPs slope was inhibited by S-Dep, which was reversed by EA treatment but not sham EA treatment. Our result is consistent with previous research, which indicated that acupuncture prevents the impairment of hippocampal LTP in vascular dementia rats [25].

Research has demonstrated that both EA treatment and pretreatment improved posttraumatic stress disorder- (PTSD-) like behaviors in rats [11, 12, 26]. Rather than PTSD, which is triggered by a traumatic event such as an aggressive incident or conflict situation [27], S-Dep-induced depression is closer to mild or minor stress-induced depression. Thus, we concluded that EA has a protective effect on either PTSD-induced major depression or S-Dep-induced minor depression.

BDNF plays a crucial role in synaptic plasticity and memory formation by targeting its high-affinity protein kinase receptor TrkB [28, 29]. Accumulating evidence addressed S-Dep-induced downregulation of BDNF in the hippocampus [30, 31]. 48 h S-Dep was associated with dramatically reduced BDNF expression in the CA1, CA3, and dentate gyrus (DG) of the hippocampus, accompanied by attenuated Ca^2+^/calmodulin-dependent protein kinase II (CAMKII) and the cAMP response element binding protein (CREB) expression and mitigated CREB phosphorylation [32, 33]. LTP is mediated by distinct signaling molecules including CAMKII and phosphorylated CREB. Downregulation of CAMKII and phosphorylated CREB impaired LTP induction [34]. This in turn would decrease the expression of key target genes including BDNF, which cycles back to mediate CREB activity [35]. In the current study, we found that EA treatment prevented S-Dep-induced attenuation of BDNF and its high-affinity receptor TrkB in the hippocampus and restored LTP induction in response; therefore, we speculated that EA might play an antidepression-like effect by regulating BDNF/TrkB signaling pathway.

## Data Availability

The data used to support the findings of this study are available from the corresponding author upon request.
